# IL18BP links antigen processing and presentation programs to a dendritic cell-enriched immunoregulatory state in colorectal cancer

**DOI:** 10.3389/fimmu.2026.1973858

**Published:** 2026-10-05

**Authors:** Junyin Zheng, Guodong Guo, Yue Tian, Liqi Chen, Haoyun Xia, Yiqing Jiang, Dajia Lin, Hao Zhang, Nan Zhang, Chenshen Huang

**Affiliations:** 1 Fuzhou University Affiliated Provincial Hospital, School of Medicine, Fuzhou University, Fuzhou, China; 2 Fujian Medical University Union Hospital, Fuzhou, China; 3 The Second Affiliated Hospital of Chongqing Medical University, Chongqing, China; 4 College of Life Science and Technology, Huazhong University of Science and Technology, Wuhan, China

**Keywords:** antigen processing and presentation, colorectal cancer, dendritic cells, IL18BP, machine learning, regulatory immune cells

## Abstract

**Background:**

Antigen processing and presentation (APP) is required for tumor immune recognition, but its relationship with the immune landscape in colorectal cancer (CRC) remains incompletely defined. In this study, APP-related transcriptional programs were used as the starting point to identify immune-cell-associated biomarkers with prognostic and immunotherapeutic relevance in CRC.

**Methods:**

Six public CRC bulk cohorts were analyzed to define APP-related molecular states. Weighted gene co-expression network analysis and four machine-learning frameworks, including Boruta, LassoLR, Pamr, and XGBoost, were applied to prioritize genes linked to APP activity. Single-cell analysis was performed with an atlas of 121 samples and 169,427 cells to identify the cellular origin of the candidate gene. Immune infiltration, cancer-immunity-cycle activity, immune checkpoint blockade (ICB) response, predicted drug response, somatic mutation patterns, and *in vitro* function were then evaluated.

**Results:**

Two APP-associated patterns were identified and showed different survival outcomes and immune landscapes. Network- and machine-learning-based screening prioritized IL18BP as the main APP-associated candidate. Higher IL18BP expression was associated with longer survival and an immune-active phenotype, including stronger antigen-presentation signals, increased immune infiltration, checkpoint-related signatures, and cancer-immunity-cycle activation. Single-cell profiling showed that IL18BP was preferentially enriched in dendritic cells (DCs), linking the APP-derived bulk signature to a DC-centered immunoregulatory compartment. Across nine external ICB-treated cohorts, IL18BP showed response-classification performance. Drug-response prediction and somatic mutation analyses identified additional features associated with IL18BP-defined groups. In CRC cell lines, IL18BP knockdown increased proliferation, migration, and invasion.

**Conclusion:**

These findings identify IL18BP as a DC-enriched, APP-associated biomarker in CRC. By connecting APP programs in bulk tumors with DC-level expression patterns at single-cell resolution, this study positions IL18BP as an APP-associated marker of a DC-enriched immune state. These findings support further evaluation of IL18BP for prognostic and immunotherapy stratification in colorectal cancer but do not establish a causal APP-DC-IL18BP pathway.

## Introduction

Colorectal cancer (CRC) remains a major cause of cancer mortality, ranking second among cancer-related deaths worldwide (1). Although surgery, chemotherapy, targeted treatment, and risk-adapted surveillance have improved disease control, metastatic spread and treatment resistance still drive many CRC-related deaths (2). Immune checkpoint blockade (ICB) has reshaped cancer therapy, but its durable benefit in CRC is concentrated in microsatellite instability-high (MSI-H) tumors. Most microsatellite-stable (MSS) tumors remain poorly responsive, which has shifted attention from checkpoint expression alone to the immune programs that determine whether an antitumor response can be generated and maintained (3). Biomarkers that capture these programs are needed to explain immune escape and to refine treatment selection.

Antigen processing and presentation (APP) provides the molecular route through which tumor-derived proteins are converted into peptide-MHC complexes recognized by T cells (4). When this machinery is weakened, tumor antigens become less visible, immune surveillance is impaired, and ICB efficacy declines (5). APP-related signatures have been associated with prognosis and immune regulation across cancers, but two gaps remain in CRC (6). First, APP variation has often been treated as a bulk-tumor score, although APP activity can arise from malignant, stromal, and immune compartments. Second, the immune-cell state that connects APP variation with therapeutic response has not been clearly resolved.

Interleukin-18 binding protein (IL18BP) is a soluble decoy receptor that binds interleukin-18 and modulates Th1-skewed inflammation and effector-cell cytotoxicity (7). Its role in cancer is context-dependent, because IL18BP can restrain inflammatory injury while also shaping cytokine-dependent antitumor immunity (8). This dual biology makes IL18BP difficult to interpret from bulk expression alone. In CRC, the key question is whether IL18BP simply reflects general inflammation or marks a more specific antigen-presenting immune compartment. Because dendritic cells (DCs) coordinate antigen presentation, T-cell priming, and immune regulation, DC-level localization is essential for interpreting an APP-linked IL18BP signal.

A bulk-to-single-cell strategy was therefore used to connect molecular stratification with cellular origin. APP-related states were first defined in bulk CRC cohorts, and a biological network algorithm was used to identify genes embedded in the APP-associated co-expression structure. Four machine-learning models then prioritized stable candidates from this network-derived gene set. This design allowed candidate selection to start from APP biology rather than from a generic prognostic screen.

IL18BP emerged from this APP-centered workflow and was then traced in a large single-cell atlas of the CRC tumor microenvironment. The resulting analysis connected IL18BP to DCs, survival, ICB-response-related transcriptional signatures, external ICB response classification, in silico drug-response patterns, somatic mutation features, and CRC cell behavior. Together, these analyses characterize an association between APP-related transcriptional programs, DC-enriched IL18BP expression, and clinically relevant immune features in colorectal cancer.

## Materials and methods

### Data collection

CRC transcriptomic profiles and matched clinicopathological data were collected from six public cohorts: TCGA-CRC, GSE106584 (9), GSE17536 (10), GSE17538 (10), GSE29621 (11), and GSE41258 (12). Single-cell analysis used seven colorectal cancer-related resources: AHCA, GutCellAtlasColon, GutCellAtlasIntestine, TabulaSapiens, TissueImmuneCellAtlas, GSE132465 (13), and GSE144735 (13). After harmonization, the atlas contained 121 samples and 169,427 cells. Single-cell RNA sequencing data were processed with Seurat (14). Core APP genes were retrieved from ImmPort.

### Single-cell data processing

The merged single-cell RNA-sequencing count matrix was processed using Seurat. Genes detected in at least three cells and cells with at least 200 detected genes were retained. Mitochondrial transcript content was calculated using the percentage of genes matching the pattern “^MT-”, and cells with a mitochondrial transcript fraction of 20% or greater were excluded. Data were normalized using the LogNormalize method with a scale factor of 10,000. The 2,000 most variable genes were selected, and percent.mt was regressed during data scaling. Principal component analysis was performed using 30 components. Harmony batch correction was applied using orig.ident as the grouping variable. UMAP, nearest-neighbor graph construction, and clustering were then performed using Harmony dimensions 1-30, and clustering resolutions from 0.1 to 1.0 were evaluated.

### Computational analysis

ConsensusClusterPlus was used to classify CRC patients into APP-associated subgroups with the partitioning around medoids algorithm (15). Differentially expressed genes (DEGs) between APP patterns were identified with limma (16). Malignant epithelial cells in the tumor microenvironment were annotated by inferCNV, and UMAP was used for dimensionality reduction and visualization. An APP-centered biological network was constructed with weighted gene co-expression analysis at a soft-thresholding power of three (17). Candidate DEGs were filtered with Boruta (18), LassoLR (19), Pamr (20), and XGBoost (21).

ESTIMATE was applied to calculate stromal score, immune score, ESTIMATE score, and tumor purity (22). IL18BP was evaluated against immunotherapy-response metrics, including CYT (23), IFNγ (24), Ayers expIS (24), T-cell-inflamed (24), Roh IS (25), Davoli IS (26), RIR (27), and ICBnetIS (28, 29). Functional enrichment was examined with Metascape and gene set enrichment analysis against Gene Ontology terms (30). Immune-cell abundance was inferred with MCPcounter (31), single-sample gene set enrichment analysis signatures described by Pornpimol (32), and TIMER (33). Single-sample gene set enrichment analysis quantified steps of the cancer-immunity cycle (34). Predicted drug response was estimated using oncoPredict (35), and somatic mutations were summarized with Maftools (36).

### *In vitro* functional studies

LOVO and RKO CRC cells were maintained in DMEM containing 10% FBS and antibiotics at 37 °C with 5% CO_2_. Cells were transfected with IL18BP-targeting siRNA or non-targeting control siRNA according to the supplier’s protocol. Proliferation was measured by EdU incorporation, and migration or invasion was measured in Transwell chambers without or with Matrigel. After 48 h, migrated or invaded cells were fixed, stained, and counted.

## Results

### APP-based molecular stratification identifies immune-state heterogeneity in CRC

APP gene expression separated CRC patients into two molecular patterns, Pattern 1 and Pattern 2 (**Figure 1A**). PCA showed transcriptomic separation between the two patterns (**Figure 1B**). Pattern 1 patients had longer overall survival than Pattern 2 patients (**Figure 1C**). Differentially expressed genes between the patterns were enriched for immune pathways, including antigen processing and presentation, chemokine signaling, cytokine-cytokine receptor interactions, NK cell-mediated cytotoxicity, and T-cell/Th-cell differentiation (**Figure 1D**). These results established APP variation as a source of clinically relevant immune-state heterogeneity in CRC.

**Figure 1:**
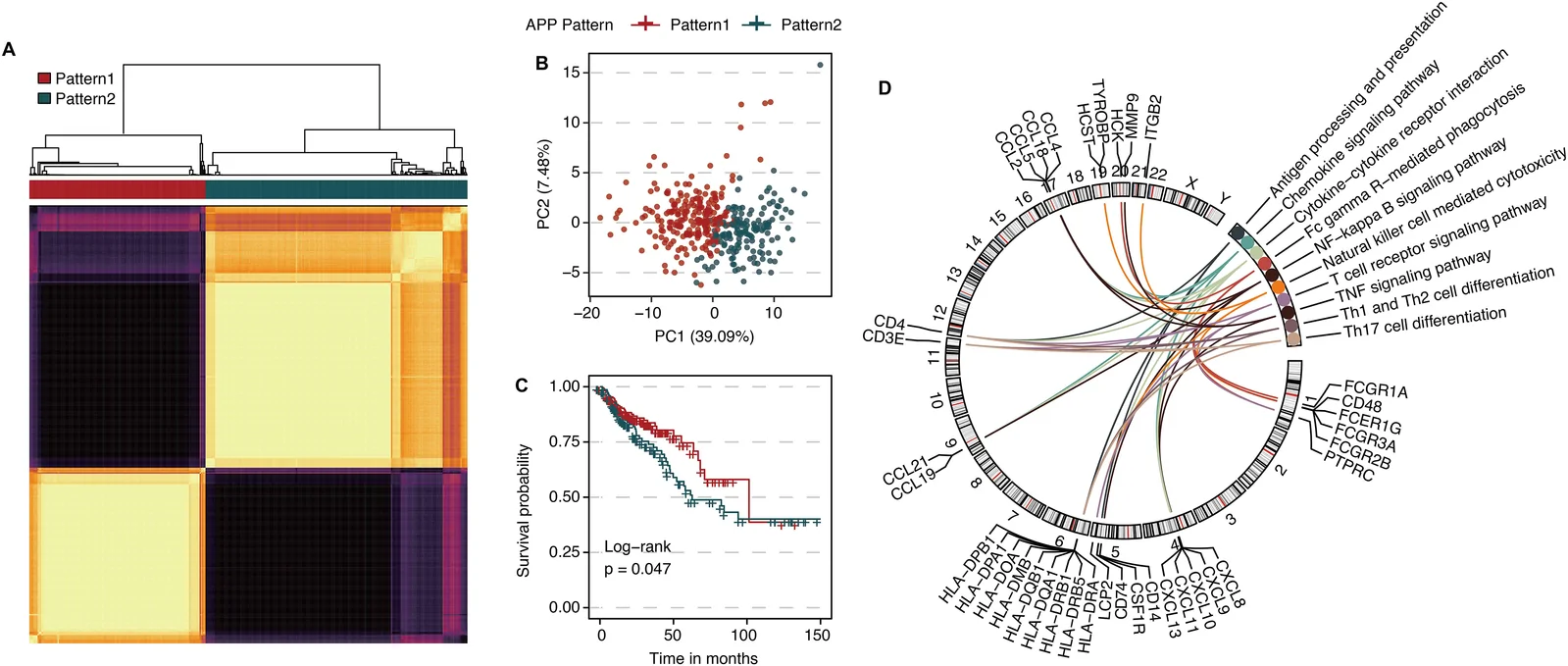
APP-related molecular patterns in CRC. **(A)** Consensus matrix for APP-related clustering. **(B)** PCA plot of the APP-defined patterns. **(C)** Overall-survival curves for the two APP patterns. **(D)** KEGG enrichment of differentially expressed genes between APP patterns. APP, antigen processing and presentation; CRC, colorectal cancer; PCA, principal component analysis; KEGG, Kyoto Encyclopedia of Genes and Genomes.

### A biological network algorithm identifies an APP-correlated gene module

A biological network algorithm was applied to move from APP patterning to candidate-gene discovery. At a soft power of 3, the network approximated scale-free topology (**Figure 2A**) and resolved several co-expression modules (**Figure 2B**). The turquoise module showed the strongest positive correlation with the APP signature (**Figure 2C**). Module membership and gene significance were positively correlated within this module (**Figure 2D**), and the inter-module network showed its position in the broader co-expression structure (**Figure 2E**). Turquoise-module genes were therefore carried forward as the APP-network-derived candidate pool.

**Figure 2:**
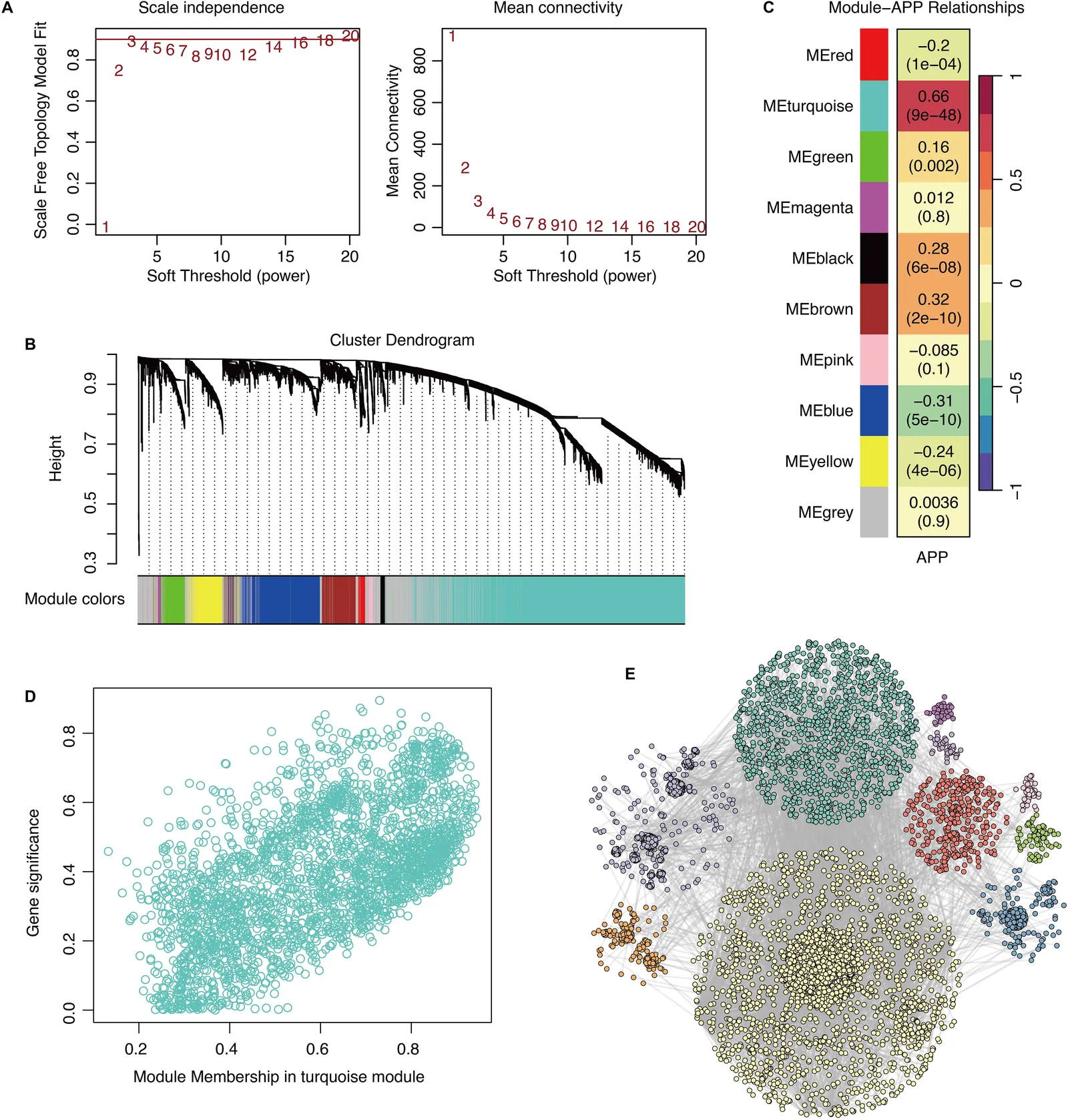
APP-associated biological network module in CRC. **(A)** Scale-free topology fit and mean connectivity across soft-power thresholds. **(B)** Distribution of identified gene modules. **(C)** Correlations between APP signature scores and gene modules. **(D)** Module membership versus gene significance in the turquoise module. **(E)** Inter-module relationships within the co-expression network.

### Multi-algorithm screening nominates IL18BP as the APP-linked candidate gene

Turquoise-module genes were intersected with differentially expressed genes from APP patterns, yielding 200 overlapping candidates (**Figure 3A**). Four feature-selection approaches, Boruta, LassoLR, Pamr, and XGBoost, converged on IL18BP, HLA-DPB1, and HLA-DMB (**Figure 3B**). IL18BP was selected for downstream analysis because it represented an immune-regulatory candidate that had not been well resolved in CRC.

**Figure 3:**
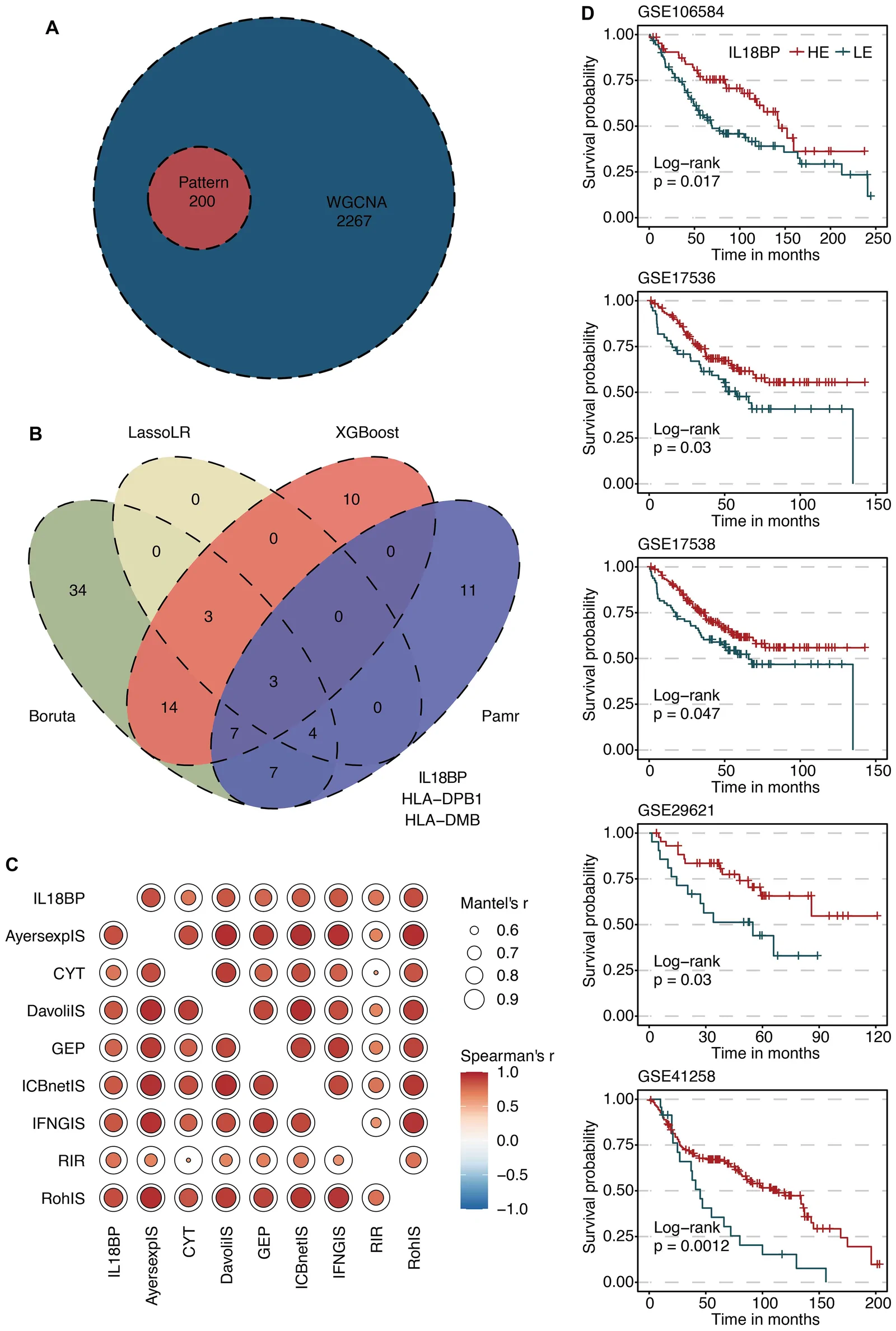
Network- and machine-learning-based selection of IL18BP. **(A)** Overlap between turquoise-module genes and differentially expressed genes from APP patterns. **(B)** Candidate genes retained by Boruta, LassoLR, XGBoost, and Pamr. **(C)** Associations between IL18BP expression and eight ICB-response-related transcriptional signatures. **(D)** Survival curves for IL18BP-defined groups in GSE106584, GSE17536, GSE17538, GSE29621, and GSE41258. ICB, immune checkpoint blockade.

IL18BP expression was positively associated with all eight ICB-response-related transcriptional signatures (**Figure 3C**). In five GEO cohorts (GSE106584, GSE17536, GSE17538, GSE29621, and GSE41258), higher IL18BP expression was associated with longer overall survival (**Figure 3D**). Thus, the APP-centered network and machine-learning workflow nominated IL18BP as a prognostic candidate associated with ICB-response-related transcriptional features.

### IL18BP silencing increases malignant phenotypes in CRC cells *in vitro*

IL18BP transcripts were detectable in LOVO and RKO cells, permitting knockdown experiments in these two CRC cell lines. RT-qPCR confirmed efficient downregulation after transfection with IL18BP-targeting siRNA (**Figures 4A, B**). Transwell assays showed increased migration and invasion after IL18BP depletion (**Figures 4C–F**). EdU assays also showed higher proliferative activity after IL18BP silencing (**Figures 4G–J**). These experiments examined a tumor-cell-associated effect of IL18BP and were not designed to identify the major cellular source of IL18BP in CRC tissue.

**Figure 4:**
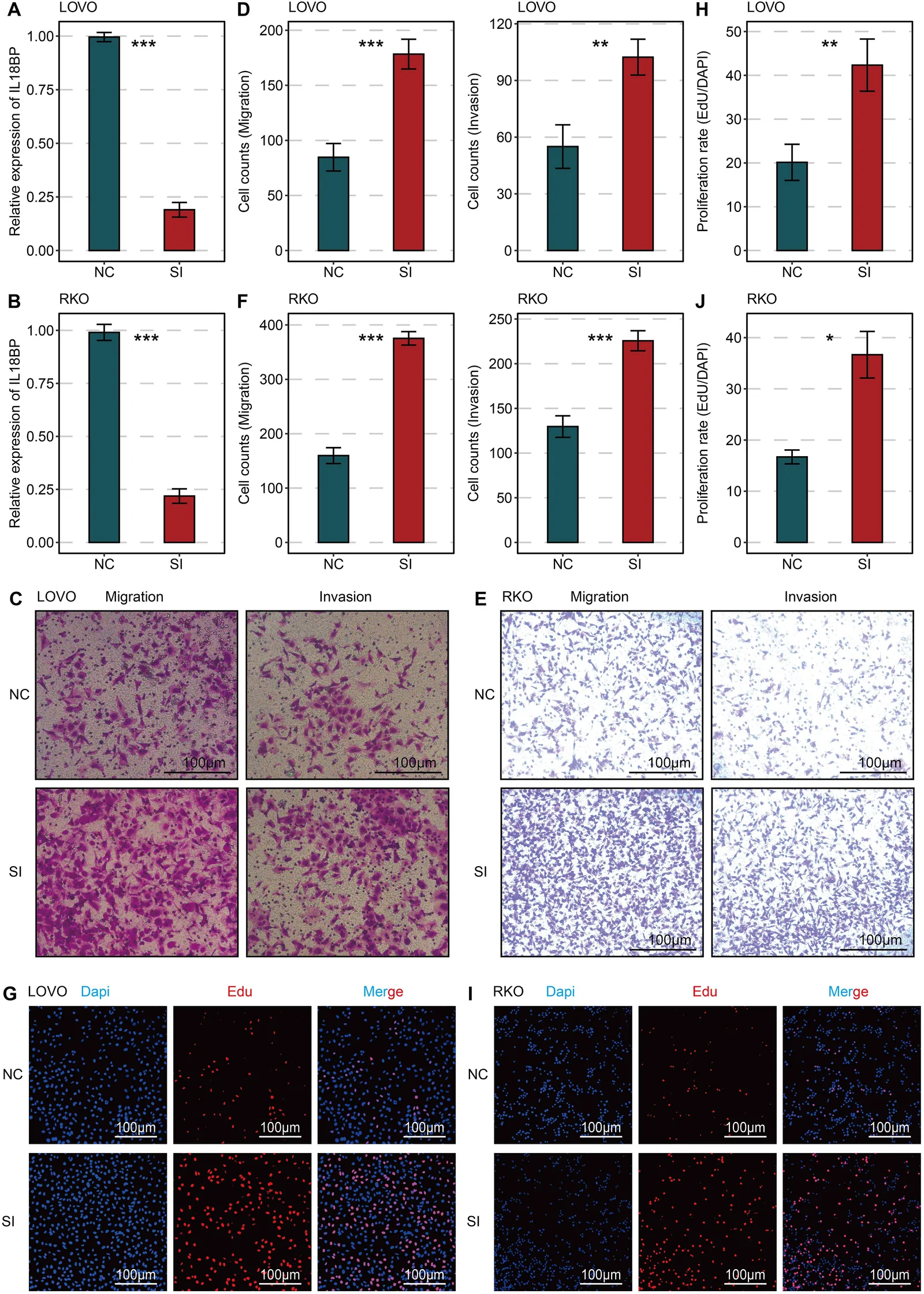
Effects of IL18BP knockdown in CRC cell lines. **(A, B)** RT-qPCR confirmation of IL18BP knockdown in LOVO and RKO cells. **(C, E)** Representative Transwell migration and invasion images for NC and siRNA-IL18BP groups in LOVO and RKO cells. **(D, F)** Quantification of Transwell migration and invasion in LOVO and RKO cells. **(G, I)** Representative EdU images for NC and siRNA-IL18BP groups in LOVO and RKO cells. **(H, J)** Quantification of EdU-positive cells in LOVO and RKO cells. *, P < 0.05; **, P < 0.01; ***, P < 0.001.

### A large single-cell atlas maps IL18BP to dendritic cells in the CRC TME

Seven scRNA-seq datasets were integrated into an atlas containing 121 samples and 169,427 cells. After quality control and clustering, cells were assigned to epithelial, stromal, and immune lineages (**Figure 5A**). Finer annotation resolved B cells, DCs, endothelial cells, fibroblasts, natural killer cells, T cells, plasma cells, and other populations (**Figure 5B**). inferCNV distinguished malignant epithelial cells by copy-number variation patterns (**Figures 5C, D**). Canonical marker genes and GO, KEGG, and WikiPathways annotation supported the cell-type assignments (**Figure 6**). This atlas provided the cellular layer needed to interpret the bulk APP-IL18BP association.

**Figure 5:**
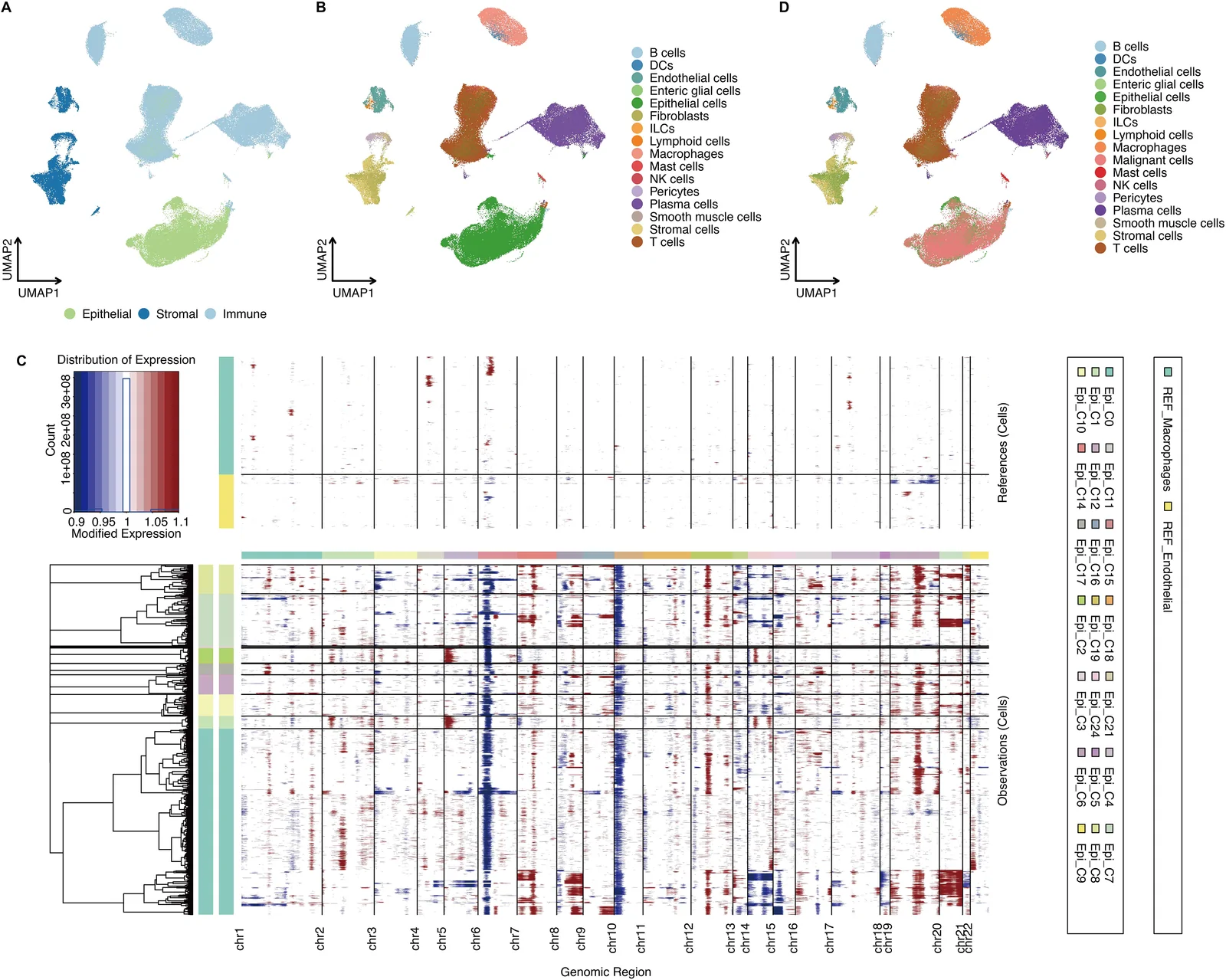
Single-cell definition of CRC TME populations. **(A)** UMAP of major TME lineages. **(B)** UMAP of finer TME cell subsets. **(C)** CNV-score heatmap across TME cells. **(D)** UMAP annotation of malignant cells. CRC, colorectal cancer; TME, tumor microenvironment; UMAP, Uniform Manifold Approximation and Projection; CNV, copy-number variation.

**Figure 6:**
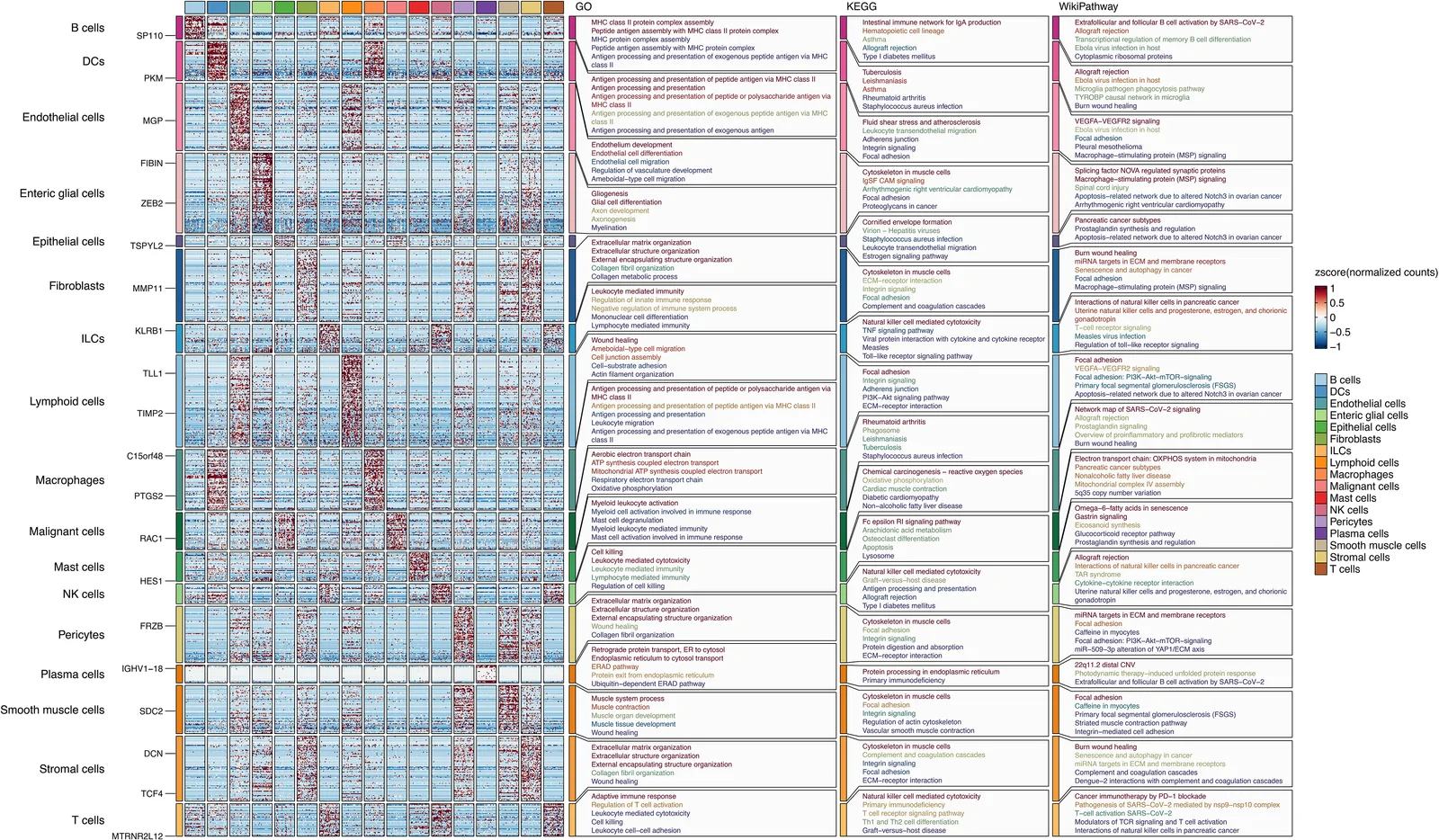
Marker-gene heatmap and functional annotation of TME cell types using GO, KEGG, and WikiPathways terms.

### IL18BP is preferentially enriched in dendritic cells and immune pathways

Across annotated populations, IL18BP expression was concentrated in DCs and remained lower in malignant and stromal compartments (**Figure 7A**). GSEA linked high IL18BP expression to immune-response programs, including inflammatory response, cytokine signaling, antigen processing and presentation, and DC/T-cell chemotaxis (**Figure 7B**). Metascape network analysis placed IL18BP-related genes in immune effector processes, positive regulation of immune responses, and cytokine signaling (**Figure 7C**). These findings suggest that the APP-associated IL18BP signal is concentrated in a DC-enriched immune compartment and is less prominent in malignant and stromal cell populations. The DC-enriched distribution observed in the single-cell atlas does not exclude lower IL18BP expression in malignant cells. Therefore, the single-cell and cell-line findings describe different biological levels of IL18BP expression in CRC.

**Figure 7:**
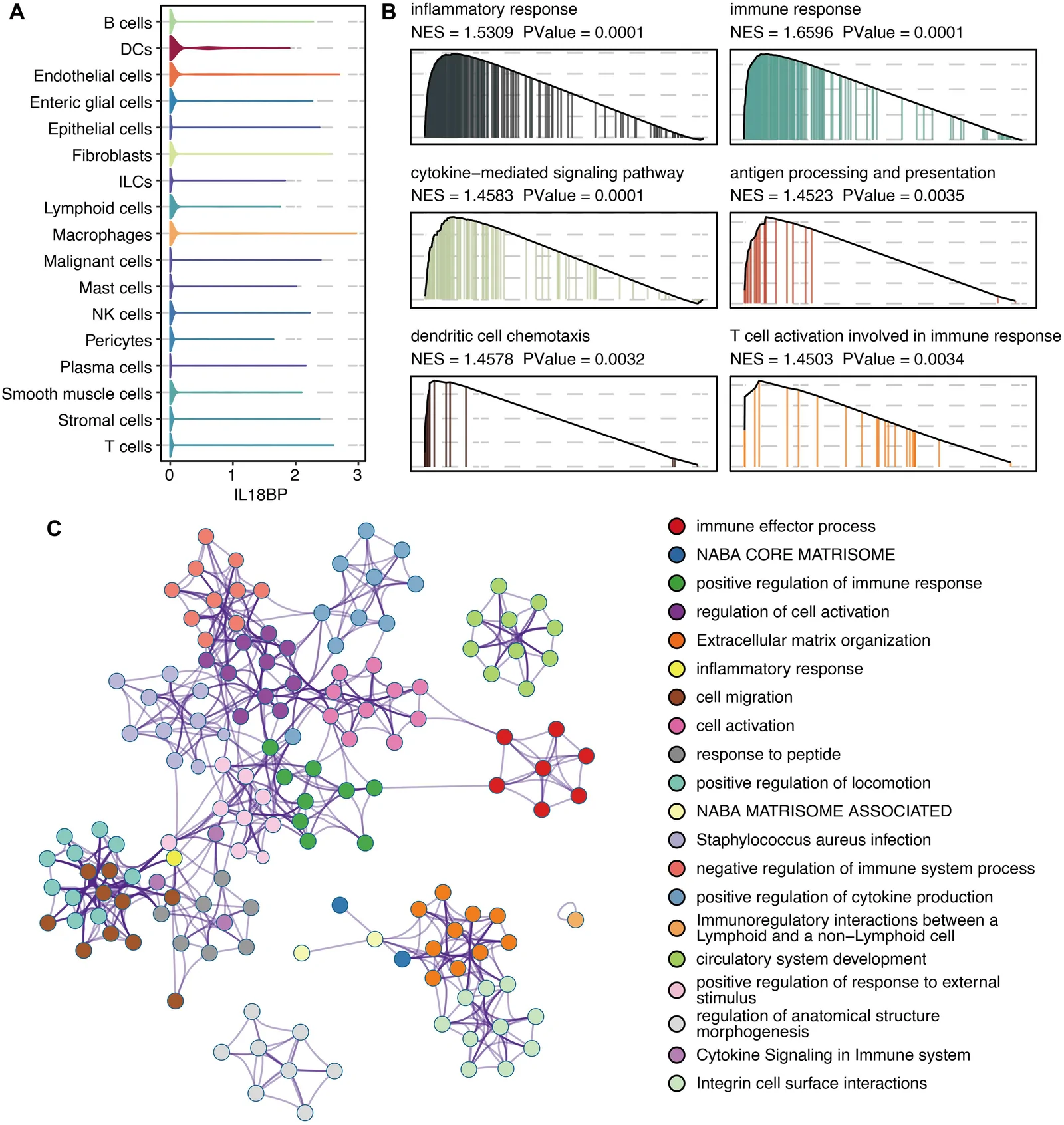
DC-enriched IL18BP expression and functional annotation. **(A)** Violin plot of IL18BP expression across TME cell types. **(B)** GO-based GSEA pathways associated with IL18BP. **(C)** Metascape pathway network for IL18BP-related genes. DCs, dendritic cells; GO, Gene Ontology; GSEA, gene set enrichment analysis; NES, normalized enrichment score.

### IL18BP marks an immune-active, cancer-immunity-cycle-high TME

IL18BP expression increased with stromal, immune, and ESTIMATE scores and decreased with tumor purity (**Figure 8A**). MCPcounter, ssGSEA, and TIMER analyses estimated higher abundance scores for T cells, CD8+ T cells, B cells, NK cells, monocytes, DCs, and neutrophils in IL18BP-high tumors (**Figure 8A**). IL18BP-high tumors also scored higher for several steps in the cancer-immunity cycle, including antigen release, antigen presentation, T-cell priming, and immune-cell recruitment (**Figure 8B**). A circos plot showed positive associations between IL18BP and immune modulators, including HLA molecules, checkpoints, chemokines, and chemokine receptors (**Figure 8C**). The bulk immune phenotype was therefore consistent with the DC-enriched single-cell signal.

**Figure 8:**
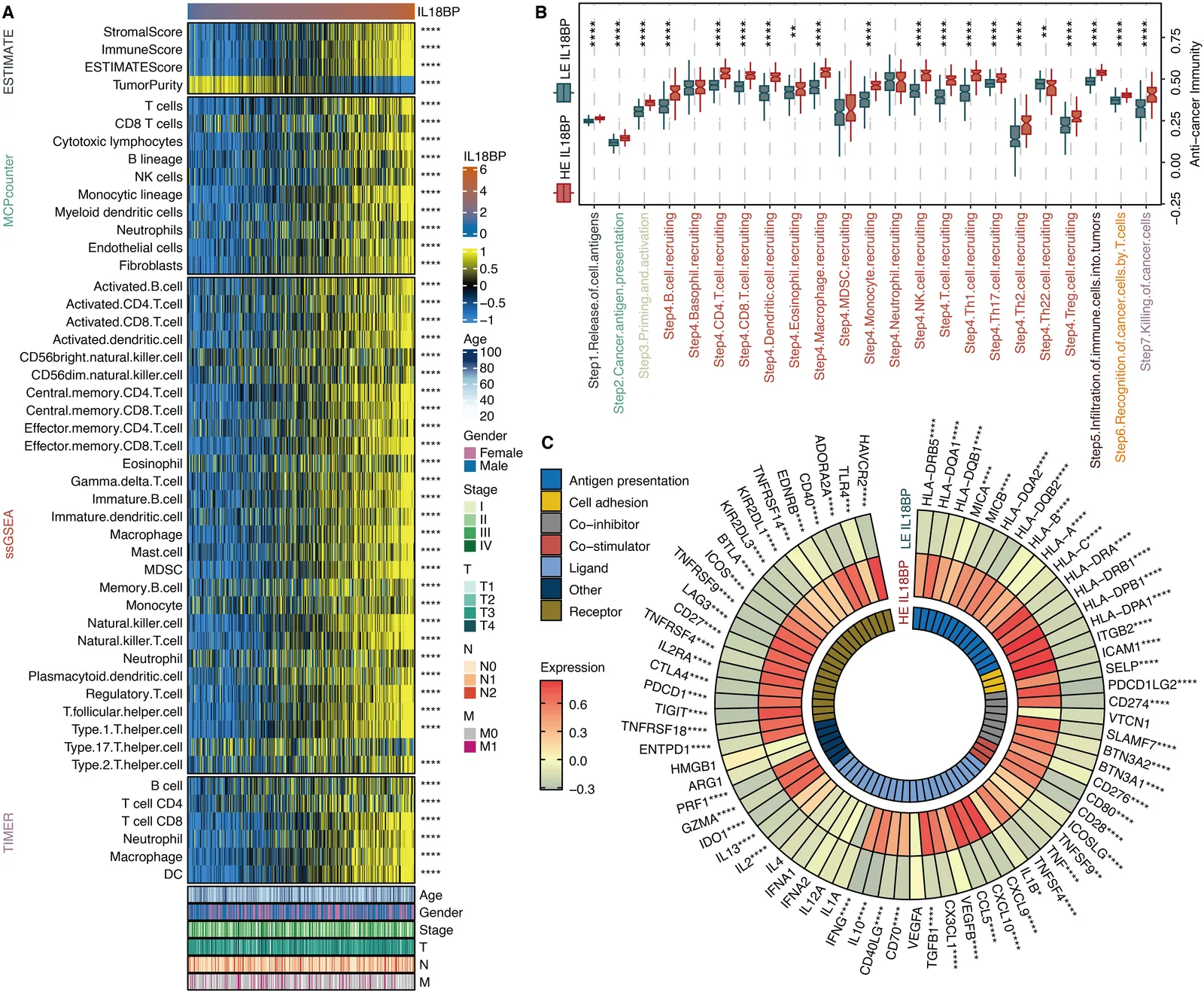
Immune features associated with IL18BP. **(A)** Correlations of IL18BP with microenvironment scores and immune-cell estimates from MCPcounter, Pornpimol-based ssGSEA, and TIMER. **(B)** Cancer-immunity-cycle scores in IL18BP-defined groups. **(C)** Circos plot linking IL18BP with immune modulators. HE, IL18BP-high expression group; LE, IL18BP-low expression group; MCPcounter, Microenvironment Cell Populations-counter; ssGSEA, single-sample gene set enrichment analysis; TIMER, Tumor Immune Estimation Resource. *, P < 0.05; **, P < 0.01; ***, P < 0.001; ****, P < 0.0001.

### IL18BP shows response-classification performance across external ICB cohorts

IL18BP was evaluated in nine external retrospective ICB-treated cohorts that included several tumor types. These datasets were used for exploratory response classification. ROC analyses showed cohort-specific response-classification performance (**Figure 9**). Overall, the cross-cohort results suggest that IL18BP captures an immune context associated with ICB response.

**Figure 9:**
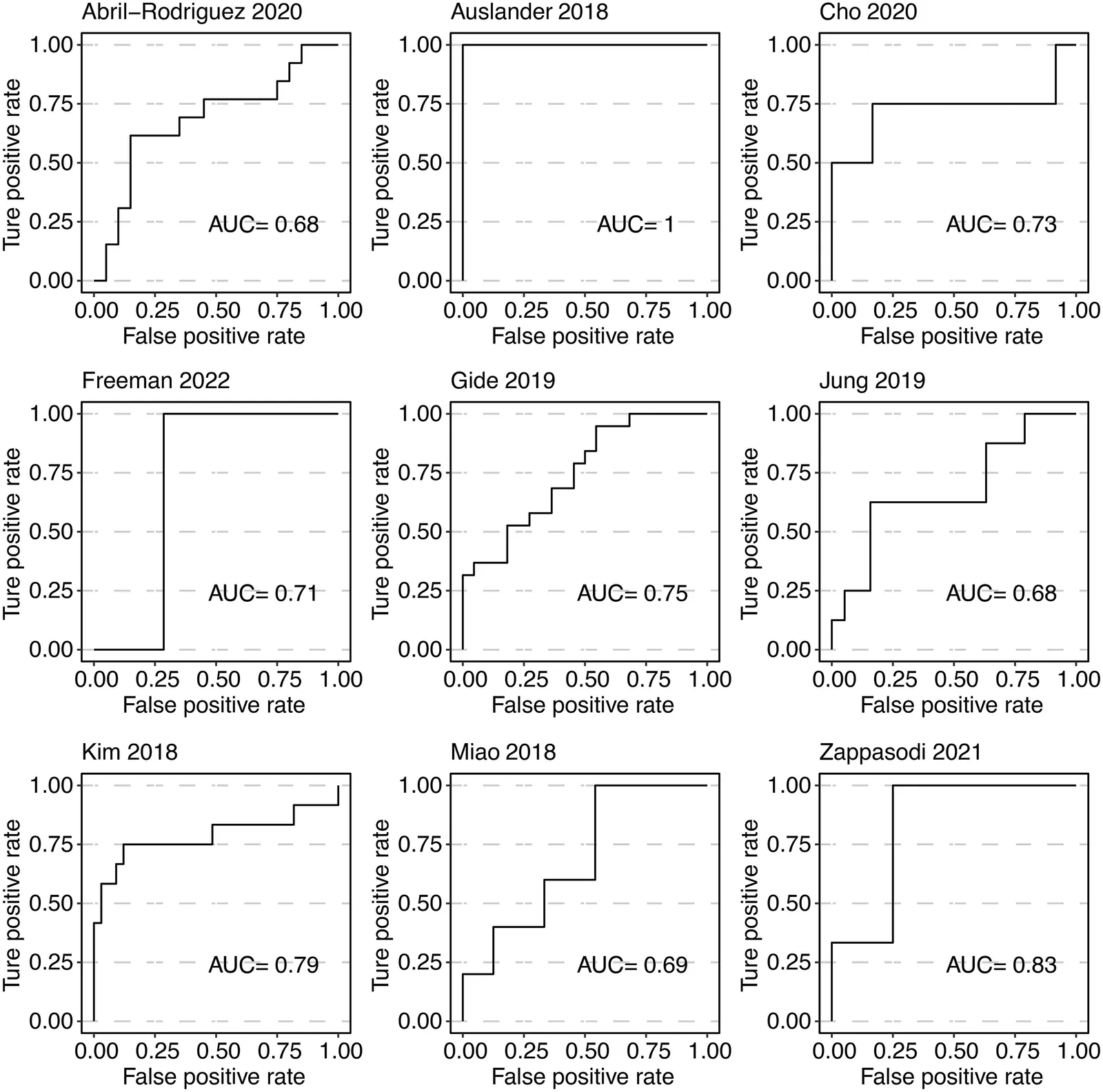
Exploratory ROC analyses of IL18BP expression for response classification in nine published external ICB-treated cohorts. Cohort-specific AUC values are shown in each panel. ROC, receiver operating characteristic; AUC, area under the curve; ICB, immune checkpoint blockade.

### IL18BP-defined groups show distinct in silico drug-response predictions

The oncoPredict was used to compare predicted drug-response scores between IL18BP-high and IL18BP-low tumors. The IL18BP-high group had higher predicted AUC values, indicating lower predicted sensitivity, for afatinib, trametinib, sapitinib, lapatinib, acetalax, ulixertinib, and osimertinib (**Figure 10**). SCH772984 and ibrutinib also showed differences between the groups. These in silico associations generate pre-clinical hypotheses and do not establish drug sensitivity, treatment resistance, or clinical benefit in patients with CRC.

**Figure 10:**
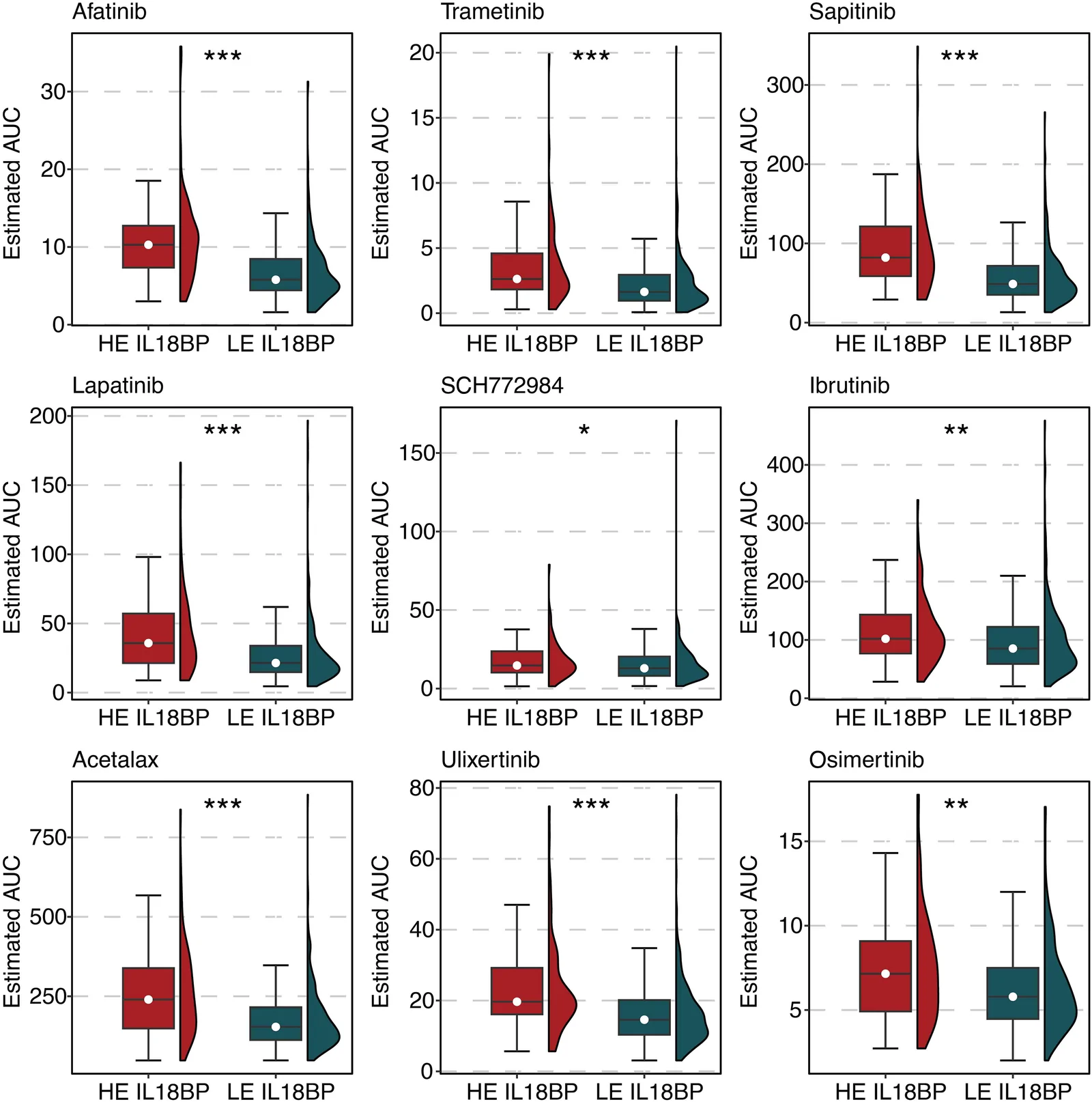
In silico predicted drug-response scores in IL18BP-defined groups. Predicted AUC values are shown for nine chemotherapy or targeted agents. Higher AUC values indicate lower predicted response. *, P < 0.05; **, P < 0.01; ***, P < 0.001.

### IL18BP expression separates mutation landscapes

Somatic mutation profiles differed between IL18BP-high and IL18BP-low tumors. APC, TP53, and KRAS appeared in both groups, whereas KRAS mutations were more frequent in the IL18BP-low group (**Supplementary Figures S1A, B**). Using Fisher’s exact test, 47 genes showed differential mutation frequencies between the IL18BP-high and IL18BP-low groups at P < 0.001 threshold. KRAS was enriched in the low-expression group, while IL18BP-high tumors more often carried mutations in POLD1, RNF213, VPS13B, NCOR1, SETD1B, and JAK1. JAK1 was among the genes enriched in the IL18BP-high group (**Supplementary Figure S2**). These mutation patterns were observed alongside IL18BP-associated immune features and should not be interpreted as consequences of IL18BP expression or as evidence of treatment response.

## Discussion

This study links APP-related transcriptional variation to a DC-enriched IL18BP signal in CRC. Bulk cohorts first separated APP states with different survival and immune landscapes. A biological network algorithm and four feature-selection models then nominated IL18BP as the stable APP-associated candidate. Single-cell mapping localized IL18BP to DCs, while cohort-level analyses connected higher IL18BP with immune infiltration, cancer-immunity-cycle activity, ICB response, and drug-sensitivity patterns. Separate CRC cell-line assays showed that IL18BP knockdown was associated with increased proliferation, migration, and invasion. These assays did not directly assess DC function, antigen processing and presentation, or T-cell priming.

APP supplies the molecular interface through which tumor antigens become visible to adaptive immunity (37). In CRC, APP-based clustering separated tumors into two molecular patterns with distinct clinical and immune profiles. Consistent with **Figure 1C**, Pattern 1 showed longer overall survival than Pattern 2. Differential expression between the two patterns was enriched for antigen presentation, cytokine signaling, and T-cell activation pathways. These pathway-enrichment results describe differences in immune transcriptional context.

IL18BP emerged as a marker associated with both APP variation and a DC-centered immune state. IL18BP binds IL-18, a cytokine that supports Th1 polarization and natural killer cell and CD8+ T-cell cytotoxicity (38). Its biological effect is therefore likely to depend on local immune context. In the CRC cohorts analyzed here, higher IL18BP expression aligned with longer survival, stronger antigen-presentation activity, and better ICB-response signatures. Single-cell data further showed preferential IL18BP expression in DCs, providing cellular context for the APP-associated bulk signal. The result suggests that IL18BP is less a general inflammatory marker than a readout of DC-centered immune organization in CRC.

The single-cell and cell-line findings should be interpreted as complementary rather than interchangeable. Single-cell profiling showed that IL18BP was preferentially enriched in DCs within the CRC tumor microenvironment, placing the main tissue-level signal in an immune context. However, preferential enrichment does not imply exclusive expression. IL18BP transcripts were also detectable in LOVO and RKO cells under the experimental conditions used here. Therefore, the siRNA assays examined the effect of IL18BP knockdown on malignant behavior in CRC cells, whereas the single-cell analysis defined the DC-enriched cellular context of IL18BP expression in tumor tissue. The cell-line experiments do not validate a DC-specific mechanism and did not directly assess antigen processing and presentation, DC function, or T-cell priming. Instead, they suggest that IL18BP may have an additional tumor-cell-associated role that is distinct from its DC-enriched expression pattern in the tumor microenvironment. Future studies using primary DCs, DC-tumor co-culture systems, and immunocompetent CRC models will be needed to test the functional relationship between IL18BP, APP activity, and antitumor immunity.

IL18BP showed response-classification performance across external ICB datasets. The marker tracked with established ICB-response-related signatures and showed favorable ROC performance across multiple cohorts. This pattern is biologically plausible because IL18BP-high tumors showed stronger immunogenicity, denser immune-cell infiltration estimates, and activated cancer-immunity-cycle steps. The single-cell enrichment of IL18BP in DCs further supports this interpretation, because DCs provide the antigen-presenting framework required for T-cell priming.

Drug-response modeling linked IL18BP status with predicted responses to several targeted agents. IL18BP-high tumors showed lower predicted sensitivity to EGFR inhibitors, including afatinib and lapatinib, and to the MEK inhibitor trametinib. These results are derived from an in silico prediction framework and should be considered hypothesis-generating. Validation in CRC models and clinically annotated treatment cohorts is needed before they can inform therapeutic selection. Prospective clinical studies and experimental validation will be required before these associations can be considered clinically actionable.

This study has limitations. Prospective, clinically annotated CRC cohorts are needed to validate the prognostic and treatment-related value of IL18BP. In addition, the present RNA-based signature is not directly interchangeable with a clinically usable protein assay. Future studies should assess the concordance between IL18BP mRNA and protein expression and determine whether immunohistochemistry can provide reproducible tissue-based evaluation in CRC. Most bulk analyses used retrospective public cohorts, and prospective CRC cohorts are needed to validate IL18BP as a prognostic and predictive biomarker. The external ICB cohorts included multiple tumor types and therefore did not provide CRC-specific prospective validation. Drug-response results were derived from in silico prediction models rather than measured patient outcomes. Mechanistic studies are also needed to define whether IL18BP directly regulates APP activity or mainly marks DC-associated immune organization. In particular, functional experiments in primary DCs or DC-tumor co-culture systems will be needed to determine whether IL18BP directly influences antigen processing, presentation, or T-cell priming. Immunocompetent CRC animal models, together with primary DC-tumor co-culture systems, may help determine whether IL18BP affects APP activity, DC function, T-cell priming, and antitumor immunity in a functional setting. Although the single-cell atlas included 121 samples and 169,427 cells, the contributing datasets differed in platform, sampling depth, and annotation granularity. Larger disease-matched single-cell cohorts will refine DC states and IL18BP-linked regulatory networks.

## Data Availability

The original contributions presented in the study are included in the article/**Supplementary Material**. Further inquiries can be directed to the corresponding author/s.

## References

[1] Mauri G Patelli G Crisafulli G Siena S Bardelli A . Tumor "age" in early-onset colorectal cancer. Cell. (2025) 188:589–93. doi: 10.1016/j.cell.2024.12.003 39919707

[2] Abedizadeh R Majidi F Khorasani HR Abedi H Sabour D . Colorectal cancer: a comprehensive review of carcinogenesis, diagnosis, and novel strategies for classified treatments. Cancer Metastasis Rev. (2024) 43:729–53. doi: 10.1007/s10555-023-10158-3 38112903

[3] Yu B Kang J Lei H Li Z Yang H Zhang M . Immunotherapy for colorectal cancer. Front Immunol. (2024) 15:1433315. doi: 10.3389/fimmu.2024.1433315 39238638 PMC11375682

[4] Pishesha N Harmand TJ Ploegh HL . A guide to antigen processing and presentation. Nat Rev Immunol. (2022) 22:751–64. doi: 10.1038/s41577-022-00707-2 35418563

[5] Yang K Halima A Chan TA . Antigen presentation in cancer - mechanisms and clinical implications for immunotherapy. Nat Rev Clin Oncol. (2023) 20:604–23. doi: 10.1038/s41571-023-00789-4 37328642

[6] Lee MY Jeon JW Sievers C Allen CT . Antigen processing and presentation in cancer immunotherapy. J Immunother Cancer. (2020) 8:e001111. doi: 10.1136/jitc-2020-001111 32859742 PMC7454179

[7] Sharma A Bishara GG Olejniczak SH Ohm JE Brentjens RJ Gupta A . Interleukin-18 armours antitumour immune effectors. Nat Rev Immunol. (2026). doi: 10.1038/s41577-026-01330-1 42463973

[8] Novick D . IL-18 and IL-18BP: A unique dyad in health and disease. Int J Mol Sci. (2024) 25:13505. doi: 10.3390/ijms252413505 39769266 PMC11727785

[9] Zhu J Deane NG Lewis KB Padmanabhan C Washington MK Ciombor KK . Evaluation of frozen tissue-derived prognostic gene expression signatures in FFPE colorectal cancer samples. Sci Rep. (2016) 6:33273. doi: 10.1038/srep33273 27623752 PMC5021945

[10] Smith JJ Deane NG Wu F Merchant NB Zhang B Jiang A . Experimentally derived metastasis gene expression profile predicts recurrence and death in patients with colon cancer. Gastroenterology. (2010) 138:958–68. doi: 10.1053/j.gastro.2009.11.005 19914252 PMC3388775

[11] Chen DT Hernandez JM Shibata D McCarthy SM Humphries LA Clark W . Complementary strand microRNAs mediate acquisition of metastatic potential in colonic adenocarcinoma. J Gastrointest Surg. (2012) 16:905–12. doi: 10.1007/s11605-011-1815-0 22362069 PMC6753785

[12] Sheffer M Bacolod MD Zuk O Giardina SF Pincas H Barany F . Association of survival and disease progression with chromosomal instability: a genomic exploration of colorectal cancer. Proc Natl Acad Sci USA. (2009) 106:7131–6. doi: 10.1073/pnas.0902232106 19359472 PMC2678450

[13] Lee HO Hong Y Etlioglu HE Cho YB Pomella V Van den Bosch B . Lineage-dependent gene expression programs influence the immune landscape of colorectal cancer. Nat Genet. (2020) 52:594–603. doi: 10.1038/s41588-020-0636-z 32451460

[14] Satija R Farrell JA Gennert D Schier AF Regev A . Spatial reconstruction of single-cell gene expression data. Nat Biotechnol. (2015) 33:495–502. doi: 10.1038/nbt.3192 25867923 PMC4430369

[15] Wilkerson MD Hayes DN . ConsensusClusterPlus: a class discovery tool with confidence assessments and item tracking. Bioinformatics. (2010) 26:1572–3. doi: 10.1093/bioinformatics/btq170 20427518 PMC2881355

[16] Ritchie ME Phipson B Wu D Hu Y Law CW Shi W . limma powers differential expression analyses for RNA-sequencing and microarray studies. Nucleic Acids Res. (2015) 43:e47. doi: 10.1093/nar/gkv007 25605792 PMC4402510

[17] Langfelder P Horvath S . WGCNA: an R package for weighted correlation network analysis. BMC Bioinf. (2008) 9:559. doi: 10.1186/1471-2105-9-559 19114008 PMC2631488

[18] Kursa MB Jankowski A Rudnicki WR . Boruta – a system for feature selection. Fundam Inform. (2010) 101:271–85. doi: 10.3233/FI-2010-288

[19] Ranstam J Cook J . LASSO regression. J Br Surg. (2018) 105:1348. doi: 10.1002/bjs.1089542587375

[20] Tibshirani R Hastie T Narasimhan B Chu G . Diagnosis of multiple cancer types by shrunken centroids of gene expression. Proc Natl Acad Sci USA. (2002) 99:6567–72. doi: 10.1073/pnas.082099299 12011421 PMC124443

[21] Chen T Guestrin C . Xgboost: a scalable tree boosting system. (2016).

[22] Yoshihara K Shahmoradgoli M Martínez E Vegesna R Kim H Torres-Garcia W . Inferring tumour purity and stromal and immune cell admixture from expression data. Nat Commun. (2013) 4:2612. doi: 10.1038/ncomms3612 24113773 PMC3826632

[23] Rooney MS Shukla SA Wu CJ Getz G Hacohen N . Molecular and genetic properties of tumors associated with local immune cytolytic activity. Cell. (2015) 160:48–61. doi: 10.1016/j.cell.2014.12.033 25594174 PMC4856474

[24] Ayers M Lunceford J Nebozhyn M Murphy E Loboda A Kaufman DR . IFN-gamma-related mRNA profile predicts clinical response to PD-1 blockade. J Clin Invest. (2017) 127:2930–40. doi: 10.1172/JCI91190 28650338 PMC5531419

[25] Roh W Chen PL Reuben A Spencer CN Prieto PA Miller JP . Integrated molecular analysis of tumor biopsies on sequential CTLA-4 and PD-1 blockade reveals markers of response and resistance. Sci Transl Med. (2017) 9:eaah3560. doi: 10.1126/scitranslmed.aah3560 28251903 PMC5819607

[26] Davoli T Uno H Wooten EC Elledge SJ . Tumor aneuploidy correlates with markers of immune evasion and with reduced response to immunotherapy. Science. (2017) 355:eaaf8399. doi: 10.1126/science.aaf8399 28104840 PMC5592794

[27] Jerby-Arnon L Shah P Cuoco MS Rodman C Su MJ Melms JC . A cancer cell program promotes T cell exclusion and resistance to checkpoint blockade. Cell. (2018) 175:984–997 e24. doi: 10.1016/j.cell.2018.09.006 30388455 PMC6410377

[28] Zhang N Yang M Yang JM Zhang CY Guo AY . A predictive network-based immune checkpoint blockade immunotherapeutic signature optimizing patient selection and treatment strategies. Small Methods. (2024) 8:e2301685. doi: 10.1002/smtd.202301685 38546036

[29] Zhang N Zhang H Li S Wu W Luo P Liu Z . Uncovering the predictive and immunomodulatory potential of transient receptor potential melastatin family-related CCNE1 in pan-cancer. Mol Cancer. (2024) 23:258. doi: 10.1186/s12943-024-02169-7 39551726 PMC11572178

[30] Zhou Y Zhou B Pache L Chang M Khodabakhshi AH Tanaseichuk O . Metascape provides a biologist-oriented resource for the analysis of systems-level datasets. Nat Commun. (2019) 10:1523. doi: 10.1038/s41467-019-09234-6 30944313 PMC6447622

[31] Becht E Giraldo NA Lacroix L Buttard B Elarouci N Petitprez F . Estimating the population abundance of tissue-infiltrating immune and stromal cell populations using gene expression. Genome Biol. (2016) 17:218. doi: 10.1186/s13059-016-1070-5 27765066 PMC5073889

[32] Charoentong P Finotello F Angelova M Mayer C Efremova M Rieder D . Pan-cancer immunogenomic analyses reveal genotype-immunophenotype relationships and predictors of response to checkpoint blockade. Cell Rep. (2017) 18:248–62. doi: 10.1016/j.celrep.2016.12.019 28052254

[33] Li T Fu J Zeng Z Cohen D Li J Chen Q . TIMER2.0 for analysis of tumor-infiltrating immune cells. Nucleic Acids Res. (2020) 48:W509–14. doi: 10.1093/nar/gkaa407 32442275 PMC7319575

[34] Chen DS Mellman I . Oncology meets immunology: the cancer-*immunity* cycle. Immunity. (2013) 39:1–10. doi: 10.1016/j.immuni.2013.07.012 23890059

[35] Maeser D Gruener RF Huang RS . oncoPredict: an R package for predicting *in vivo* or cancer patient drug response and biomarkers from cell line screening data. Brief Bioinform. (2021) 22:bbab260. doi: 10.1093/bib/bbab260 34260682 PMC8574972

[36] Mayakonda A Lin DC Assenov Y Plass C Koeffler HP . Maftools: efficient and comprehensive analysis of somatic variants in cancer. Genome Res. (2018) 28:1747–56. doi: 10.1101/gr.239244.118 30341162 PMC6211645

[37] Mpakali A Stratikos E . The role of antigen processing and presentation in cancer and the efficacy of immune checkpoint inhibitor immunotherapy. Cancers (Basel). (2021) 13:134. doi: 10.3390/cancers13010134 33406696 PMC7796214

[38] Park SY Hisham Y Shin HM Yeom SC Kim S . Interleukin-18 binding protein in immune regulation and autoimmune diseases. Biomedicines. (2022) 10:1750. doi: 10.3390/biomedicines10071750 35885055 PMC9313042

