# Supplementary material for: IL18BP links antigen processing and presentation programs to a dendritic cell-enriched immunoregulatory state in colorectal cancer
Source: Front Immunol. 2026 Oct 5;17:1973858. doi: 10.3389/fimmu.2026.1973858 (PMC13677846; doi:10.3389/fimmu.2026.1973858)
Supplement: Supplementary file 1 [file DataSheet1.docx]

Figure S1. Somatic mutation landscapes stratified by IL18BP expression. A. Waterfall plot showing the top mutated genes in the IL18BP-high group. B. Waterfall plot showing the top mutated genes in the IL18BP-low group. The upper bars indicate tumor mutation burden for each sample, and mutation types are annotated by color.

Figure S2. Differentially mutated genes between IL18BP-low and IL18BP-high groups. Forest plot showing odds ratios and 95% confidence intervals for 47 genes with different mutation frequencies between the two IL18BP-defined groups. Mutation frequencies were compared using two-sided Fisher’s exact tests. Genes shown met P < 0.001 threshold. Odds ratios below 1 indicate enrichment in the IL18BP-low group, whereas odds ratios above 1 indicate enrichment in the IL18BP-high group.
